# Vaccination and Small-Pox

**Published:** 1895-07

**Authors:** D. N. Banerjee

**Affiliations:** Calcutta, India


### VACCINATION AND SMALL-POX.

Sir The Commandeur, Dr. D. N. Banerjee, Calcutta, India.

I should like if the editor will very kindly afford me a piece of help by ventilating this subject in his esteemed and widely circulated journal. It was originally published through the medium of a local paper of this town:

"Sir: During this change of season pox of different kinds is breaking out in different localities, which we must accept as the will of God. Does any physician think for a moment that vaccination or re-vaccination or any preventive medicine can check or prevent its appearance or its course? I would, therefore, advise every one not to eat or drink anything which is heat-given, and to keep the health as much as possible free from atmospheric poison floating in the air by warm clothing. I had several cases of small-pox this year, and I got them cured simply by suitable diet (as milk with sago) and without medicine, and the disease has never broken out in an epidemic form in their families. These patients were vaccinated in their infancy with the lymph of the cow, and I can point out many cases in which men are in ripe age and in sound health without vaccination."

I also beg to inform your numerous readers that during the last three or four months the rate of mortality from small-pox in this town is 125 to 150 per week, and they are, I am sure, all vaccinated, as the laws of this country compel them to be. I therefore beg that our government will abolish the practice of vaccination, and that the laws concerning it be repealed.

